# Impact of the Marker Set Configuration on the Accuracy of Gait Event Detection in Healthy and Pathological Subjects

**DOI:** 10.3389/fnhum.2021.720699

**Published:** 2021-09-13

**Authors:** Rosa M. S. Visscher, Marie Freslier, Florent Moissenet, Sailee Sansgiri, Navrag B. Singh, Elke Viehweger, William R. Taylor, Reinald Brunner

**Affiliations:** ^1^Laboratory for Movement Biomechanics, Department of Health Science and Technology, Institute for Biomechanics, ETH Zürich, Zurich, Switzerland; ^2^Biomechanics of Movement Group, Department of Biomedical Engineering, University of Basel, Basel, Switzerland; ^3^Laboratory for Movement Analysis, Department of Orthopedics, University Children’s Hospital Basel, Basel, Switzerland; ^4^Laboratory for Kinesiology, University of Geneva and Geneva University Hospitals, Geneva, Switzerland; ^5^Department of Biomedical Engineering, Faculty of Mechanical, Maritime, and Materials Engineering, Delft University of Technology, Delft, Netherlands

**Keywords:** gait analysis, pathological gait, cerebral palsy, gait event detection, motion capture

## Abstract

For interpreting outcomes of clinical gait analysis, an accurate estimation of gait events, such as initial contact (IC) and toe-off (TO), is essential. Numerous algorithms to automatically identify timing of gait events have been developed based on various marker set configurations as input. However, a systematic overview of the effect of the marker selection on the accuracy of estimating gait event timing is lacking. Therefore, we aim to evaluate (1) if the marker selection influences the accuracy of kinematic algorithms for estimating gait event timings and (2) what the best marker location is to ensure the highest event timing accuracy across various gait patterns. 104 individuals with cerebral palsy (16.0 ± 8.6 years) and 31 typically developing controls (age 20.6 ± 7.8) performed clinical gait analysis, and were divided into two out of eight groups based on the orientation of their foot, in sagittal and frontal plane at mid-stance. 3D marker trajectories of 11 foot/ankle markers were used to estimate the gait event timings (IC, TO) using five commonly used kinematic algorithms. Heatmaps, for IC and TO timing per group were created showing the median detection error, compared to detection using vertical ground reaction forces, for each marker. Our findings indicate that median detection errors can be kept within 7 ms for IC and 13 ms for TO when optimizing the choice of marker and detection algorithm toward foot orientation in midstance. Our results highlight that the use of markers located on the midfoot is robust for detecting gait events across different gait patterns.

## Introduction

3D Clinical Gait Analysis (CGA) is increasingly used for quantifying gait deviations, informing clinical decision making, and monitoring the effectiveness of therapy in movement disorders **(**Cimolin and Galli, 2014**;** Armand et al., 2016**)**. Accurate estimation of gait events, such as initial contact (IC) and toe-off (TO), is essential for interpreting CGA outcomes, as incorrect identification of gait events could lead to errors in normalization of kinematic and kinetic data and its ensemble-averaging, as well as to inaccurate spatio-temporal parameters. The gold standard for estimating gait event timing is the use of vertical ground reaction forces (vGRFs), during which a threshold is set, when the vGRF crossed the force threshold a gait event is detected (Bruening and Ridge, 2014). However, for this method to work in an automatic manner, clean force plate hits (during which only one foot contacts a force plate) are necessary. These clean hits are often limited due to use of assistive devices, short step lengths or partial contact with the plate, which limits the number of strides that can be used for analysis. As an alternative to vGRFs, kinematics-based algorithms have been introduced. These algorithms use the position, velocity, or acceleration of IMU sensor(s) (placed on the hip, shank, or feet) or a set of reflective cutaneous markers (placed on bone-landmarks of the foot/ankle) to estimate gait event timing. Over the years, numerous algorithms have been developed, based on various marker sets as input **(**Stanhope et al., 1990**;** Bruening and Ridge, 2014**)**. Here, previous work has shown that adaptation of the markers’ locations could improve the estimation of gait event timing, especially in pathological populations such as children with cerebral palsy (CP) (Visscher et al., 2021). Within the CP population, multiple types of pathological gait patterns are present such as True equinus, Jump knee, Apparent equinus, and Crouch gait (Rodda and Graham, 2001). Per gait pattern, the part of the foot used for IC and TO can differ. It might therefore be of interest to evaluate how different marker locations perform when different parts of the foot are initially contacting or leaving the ground. However, a systematic overview of the effect of the markers’ locations on the accuracy of estimating gait event timing is lacking.

With the aim to guide the choice of markers for optimal estimation of gait event timing, the goal of this study was to present a detection error map (with vGRFs as the reference), examining the effect of the markers’ locations on estimation of gait event timing, based on a set of commonly used kinematics-based algorithms.

## Methods

### Participants

104 individuals with CP and 31 typically developing (TD) controls were retrospectively included in this study (Table 1). All participants underwent CGA in a local hospital between August 2016 and May 2019. Each subject was categorized into 1 of 4 groups for IC estimations depending on the orientation of their foot in the sagittal plane (ankle dorsiflexion/plantarflexion) at midstance (Criteria were set based on the mean values of the TD group with thresholds chosen to allow for homogeneous distribution of patients between groups, Group IC1: >mean + 2SD *n*_TD_ = 62 median dosiflexion angle = 17.8° *n*_CP_ = 51, 9.8°; Group IC2: mean-2SD ≤ mean + 2SD *n*_TD_ = 0, *n*_CP_ = 25, -0.2°; Group IC3: mean-10SD ≤ mean-2SD *n*_TD_ = 0, *n*_CP_ = 42, -5.6°; Group IC4: <mean-10SD *n*_TD_ = 0, *n*_CP_ = 39, -15.5°; Supplementary Table 1), and into 1 of 4 groups for TO estimations depending on the orientation of their foot in the frontal plane (ankle eversion/inversion) at midstance (criteria were set based on the mean values of the TD group with thresholds chosen to allow for homogeneous distribution of participants between groups, Group TO1: >mean + 2SD *n*_TD_ = 0, *n*_CP_ = 15, 17.0°; Group TO2: mean-2SD ≤ mean + 2SD *n*_TD_ = 11, 4.3°, *n*_CP_ = 55, 5.6°; Group TO3: mean-4SD ≤ mean-2SD *n*_TD_ = 30, -2.4°, *n*_CP_ = 36, -1.8°; Group TO4: <mean-4SD *n*_TD_ = 21, -9.7°, *n*_CP_ = 50, -12.5°; Supplementary Table 1). Further information on grouping and calculation of the foot orientation can be found in Supplementary Table 1 and on our GitLab page.^1^ All participants or their guardians, gave their written informed consent prior to inclusion in the study, which as approved by the local ethical committee (2018-01449). All measurements were conducted according to the Declaration of Helsinki.

**TABLE 1:** Descriptive statistics of the participants (independent samples *T*-test, chi-square test for gender).

	TD (*n* = 31)	CP (*n* = 104)	*p*-value
Age (years)	20.6 (7.8)	16.0 (8.6)	**0.008**
Gender	15 males 16 females	66 males 38 females	0.133
Height (m)	1.64 (0.17)	1.52 (0.20)	**0.002**
Weight (kg)	55.0 (15.6)	45.9 (18.1)	0.012
BMI (kg/m^2^)	20.0 (2.80)	19.0 (4.0)	0.232
Legs evaluated (*n*)	62	156	NA
Types of CP	NA	52 Hemiplegic 52 Diplegic	NA
GMFCS	NA	I: 73 II: 31	NA

*All parameters reported as mean (SD). TD, typically developing controls; CP, cerebral palsy; SD, standard deviation; BMI, body mass index; NA, not applicable. Bold values indicate significant difference between TD and CP groups.*

### Measurement Procedure

**FIGURE 1:**
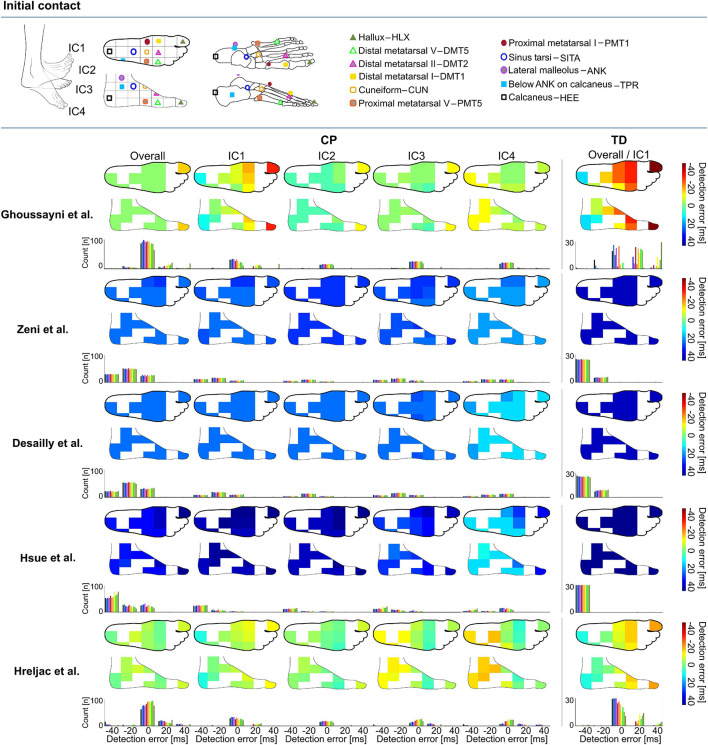
Heatmaps showing median detection errors for estimating the timing of initial contact (IC) for individuals with cerebral palsy (CP) and typical development (TD) per selected marker and method. The location of the markers is indicated on top of the figure, each marker location is connected to a pixel on the heatmap. The color of the heatmap indicates the median detection error. The histogram below each heatmap indicates the number of individuals for which a certain detection error was obtained. Groups IC1–IC4 were based on angle of the foot in the sagittal plane during midstance, Supplementary Table 1.

**FIGURE 2:**
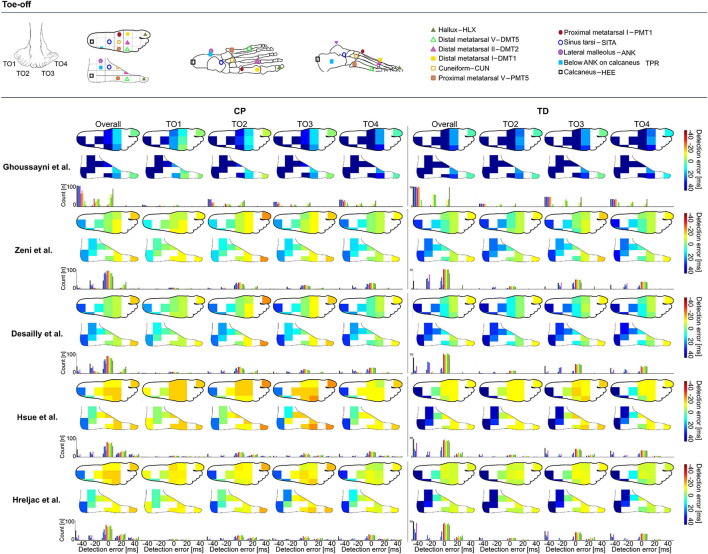
Heatmaps showing median detection errors for estimating the timing of toe-off (TO) for individuals with CP and TD. The location of the markers is indicated on top of the figure, each marker location is connected to a pixel on the heatmap. The color of the heatmap indicates the median detection error. The histogram below each heatmap indicates the number of individuals for which a certain detection error was obtained. Groups TO1–TO4 were based on angle of the foot in the sagittal plane during midstance, Supplementary Table 1.

All participants walked barefoot on an instrumented walkway at their self-selected walking speed for six trials, one trial per participant was selected for analysis. Out of a total of 64 markers (9.5 mm diameter), which were attached to the participants according to the modified Conventional Gait Model (CGM1.0) model (Kadaba et al., 1990) (Supplementary Presentation 1), 11 markers located on the foot/ankle were selected for analysis (hallux, HLX; distal metatarsal I, DMT1; distal metatarsal II, DMT2; distal metatarsal V, DMT5; proximal metatarsal I, PMT1; cuneiform, CUN; proximal metatarsal V, PMT5; sinus tarsi, SITA; lateral malleolus, ANK; below ANK on calcaneus, TPR; calcaneus, HEE; Figures 1, 2). Marker trajectories were collected at a sampling frequency of 150 Hz using a 12-camera optoelectronic motion capture system (MTX20, VICON, Oxford, United Kingdom).

### Data Processing

Data pre-processing was performed within the VICON-NEXUS software (v1.8.5), filtering was done using the built-in Woltring filter (mean squared error set to 10 mm^2^), marker gaps smaller than five frames were filled using the built-in gap filling functions. To extract gait events (IC, TO) using vGRFs, a force threshold of 20 N was applied to the vertical component of the ground reaction force signal collected through the force plates (Kistler, Switzerland, sampling frequency 1,500 Hz) embedded in the walkway (Visscher et al., 2021). Post-processing analyses were performed in MATLAB (R2019a, The Mathworks, Natick, United States) using the open-source Biomechanical ToolKit package (BTK) (Barre and Armand, 2014). Trials with excessive soft tissue artifacts, poor consistency, or signs of inaccurate marker placement were excluded after visual inspection. One valid gait cycle (GC) of each limb per participant was used in further analyses. Only the paretic side was included for hemiplegic patients.

The 3D marker trajectories of 1 of the following 11 foot/ankle markers (HLX, DMT1, DMT2, DMT5, PMT1, CUN, PMT5, SITA, ANK, TPR, and HEE, Figures 1, 2) were used to calculate the gait event timings (IC, TO) according to five previously published kinematics-based algorithms: Zeni et al. (2008), Desailly et al. (2009), Ghoussayni et al. (2004), Hsue et al. (2007), and Hreljac and Marshall (2000), Table 2 and all available on a published repository (see text footnote 1). The choice of these algorithms was guided by a previous review article that evaluated automated event detection algorithms in pathological gait (Bruening and Ridge, 2014). To reduce the chance of false positives, a time window of ±30 frames (0.2 s, as we measured at 150 Hz) around the estimated event timing by Zeni et al. (2008) was used for applying the other algorithms. The differences (in ms) in gait event timings obtained using vGRFs vs kinematics-based algorithms were defined as the detection error.

**TABLE 2:** Definition of gait events (IC, TO) according to included algorithms.

Authors	Description of IC	Description of TO	Markers used for IC and TO determination
Zeni et al., 2008	Maximum horizontal marker position relative to sacrum	Minimum horizontal marker position relative to sacrum	HLX, DMT5, DMT2, DMT1, CUN, PMT5, PMT1, SITA, ANK, TPR, and HEE
Desailly et al., 2009	High pass filtered maximum horizontal marker position (Cut-off frequency set at 0.5* cadence)	High pass filtered minimum horizontal marker position (Cut-off frequency set at 0.5* cadence)	HLX, DMT5, DMT2, DMT1, CUN, PMT5, PMT1, SITA, ANK, TPR, and HEE
Ghoussayni et al., 2004	IC occurs when the sagittal velocity of the marker falls below a threshold of 500 m/s	TO occurs when the sagittal velocity of the marker crosses a threshold of 500 m/s	HLX, DMT5, DMT2, DMT1, CUN, PMT5, PMT1, SITA, ANK, TPR, and HEE
Hsue et al., 2007	Minimum of the horizontal acceleration of the marker	Maximum of the horizontal acceleration of the marker	HLX, DMT5, DMT2, DMT1, CUN, PMT5, PMT1, SITA, ANK, TPR, and HEE
Hrejac et al., 2000	IC occurs at the local maxima in the vertical acceleration of the marker and the point of zero-crossing of the jerk (as it decreases)	TO occurs at the local maxima in the horizontal acceleration of the marker and the point of zero-crossing of the jerk (as it increases)	HLX, DMT5, DMT2, DMT1, CUN, PMT5, PMT1, SITA, ANK, TPR, and HEE

*For convenience, the algorithms have been listed according to the primary author’s last names.*

*HLX, hallux marker; DMT5, distal metatarsal V; DMT2, distal metatarsal II; DMT1, distal metatarsal I; CUN, cuneiform; PMT5, proximal metatarsal V; PMT1, proximal metatarsal I; SITA, Sinus tarsi; ANK, lateral malleolus; TPR, below ANK on calcaneus; HEE, Calcaneus.*

### Statistical Analysis

As errors were not normally distributed, the median detection error and the 95 percentile confidence interval lower and upper boundaries (95%CI) were calculated for each event (IC and TO), method (Ghoussayni et al., 2004; Zeni et al., 2008; Desailly et al., 2009; Hsue et al., 2007; Hreljac and Marshall, 2000), group (IC1–IC4 and TO1–TO4), and marker (HLX, TOE/DMT1, DMT5, PMT1, CUN, PMT5, SITA, ANK, TPR, and HEE). Median detection errors were used to create a heatmap of the top and side views of the foot for each event, method, and group. In addition, histograms were generated to indicate the number of individuals for which a certain detection error was obtained.

## Results

### Initial Contact

Depending on the choice of algorithm and marker, differences for detecting IC timings were up to 147 ms in TD and 80 ms in CP (Supplementary Data Sheet 1). For estimating the timing of IC across groups, the best estimations were obtained when using the algorithm proposed by Hreljac et al. with SITA marker for TD (median detection error: -1 ms, 95%CI: -12 to 16 ms, ±3 frames, Figure 1), and the algorithm proposed by Ghoussayni et al. with SITA or PMT5 marker for CP (median detection error: 0 ms, 95%CI: -27 to 31 ms, ± 5 frames, Figure 1). Outcomes for IC1 were most sensitive toward the marker selected for analyses when the Ghoussayni et al. approach was used, yielding differences of up to Δ80 ms in TD and Δ20 ms in CP (Figure 1, Supplementary Data Sheet 1). While in IC1 best results were achieved with rare- or mid-foot markers (HEE/TPR/ANK/SITA/PMT5), in IC4 best results were achieved with fore-foot markers (PMT1/CUN/DMT1/DMT2/DMT5/HLX). As indicated by the green pixels in Figure 1, median detection errors could be kept below 7 ms for IC for all groups when using the approaches from Ghoussayni et al. or Hreljac et al. with PMT1, CUN, or PMT5 marker (Supplementary Data Sheet 1).

### Toe-Off

Similar to the detection of IC timings, differences for detecting TO timings were up to 153 ms in TD and 143 ms in CP (Supplementary Data Sheet 1), depending on the algorithm and marker selected for analysis. For estimating the timing of TO across groups, best estimations were obtained when using the algorithm proposed by Desailly et al. with DMT5 marker in TD (median detection error: 0 ms, 95%CI: -13 to 13 ms, ±2 frames, Figure 2) and with CUN marker in CP (median detection error: 0 ms, 95%CI: -33 to 27 ms, ±6 frames, Figure 2). As indicated by the dark blue to orange heatmap pixels in Figure 2, outcomes for all groups and methods showed to be sensitive toward marker selection (Supplementary Data Sheet 1) (Δ 37–133 ms in TD, Δ 10–120 ms in CP). For TO estimation, the green pixels in Figure 2 indicate median detection errors could be kept below 13 ms for all groups when using the approaches from Zeni et al. or Desailly et al. with PMT1, CUN, or PMT5 marker (Supplementary Data Sheet 1).

## Discussion

The aim of this manuscript was to provide a systematic overview of the effects that marker selection has on the accuracy of gait event timing estimation. Our results present a median detection error map (with vGRFs as reference) according to the markers used as inputs for a set of common kinematics-based algorithms for gait event timing estimation. This map allows for ease in visualization and can be used by researchers and clinicians to optimize their choice for algorithm and marker selection depending on their participant’s gait patterns.

To our knowledge, this is the first study that has evaluated the impact of marker location on the accuracy of gait event timing estimation. Our results support the hypothesis in Visscher et al. (2021) that event detection in pathologies could be improved through optimal selection of the marker set according to the study cohort pathology. For example, generally the HEE-marker with Hreljac approach is used for estimating IC. In a TD child who presents a clear heel strike this leads to a small under estimation with a median detection error of -12 ms compared to force plate detection, however, in a spastic CP patient who presents equinus gait and uses the fore-foot for IC this could lead to an over estimation with a median detection error of +18 ms. When for both subjects the ideal method and marker is used according to the provided heatmaps and the position of their foot during midstance, the detection error can stay below 7 ms in both cases. The results of our study show that the use of markers on the midfoot (PMT1, PMT5, and CUN) is generally robust for detecting gait events across different gait patterns when using the Hreljac approach as errors for all groups stayed below 20 ms. Indicating, that clinical gait laboratories that measure a lot of different types of gait patterns and want to keep the amount of adaptations needed per case to a minimum, might benefit from including mid-foot markers to their marker model for estimating IC and TO. However, many of the frequently used marker sets in clinical practice do not include markers on the midfoot (Deschamps et al., 2011).

We acknowledge our study had some limitations. First, as the study is conducted retrospectively it has an inferior level of evidence compared to a prospective study design. Second, significant differences were observed between TD and CP cohorts for age and height, these differences might have interfered with the results. However, as the results for subgroups in TD (IC1, TO2, TO3, and TO4) are very similar to the results from the subgroups with CP, we expect the interference to be minimal. In addition, the generalizability of the study is limited as only individuals with one specific pathology (spastic cerebral palsy), walking barefoot, were included, while severely affected individuals were excluded. Further investigations should certainly expand this analysis to different pathologies and severities in order to present more global recommendations on marker selection. To support others to apply our method to their datasets, we have shared all our analysis codes and metadata on a published repository (see text footnote 1).

Overall, our study showed that gait event timing estimation can be improved by optimizing the marker set and the detection algorithm toward specific gait patterns. Our heatmaps presented in this work can be used to guide researchers and clinicians toward which marker set would be most beneficial for their intended use. Further research can expand the current approach toward other gait patterns and walking conditions.

## Data Availability Statement

The datasets presented in this study can be found in online repositories. The names of the repository/repositories can be found in the article/Supplementary Material.

## Ethics Statement

The studies involving human participants were reviewed and approved by KEK - Local ethical committee canton Zurich, Zurich, Switzerland. Written informed consent to participate in this study was provided by the participants’ legal guardian/next of kin.

## Author Contributions

RV, MF, FM, and RB contributed the conception and design of the study. RV, MF, FM, and SS developed the codes for data analysis. MF and SS processed and organized the data. RV and MF performed the statistical analysis. RV wrote the first draft of the manuscript. MF wrote sections of the manuscript. All authors contributed to manuscript revision, read, and approved the submitted version.

## Conflict of Interest

The authors declare that the research was conducted in the absence of any commercial or financial relationships that could be construed as a potential conflict of interest.

## Publisher’s Note

All claims expressed in this article are solely those of the authors and do not necessarily represent those of their affiliated organizations, or those of the publisher, the editors and the reviewers. Any product that may be evaluated in this article, or claim that may be made by its manufacturer, is not guaranteed or endorsed by the publisher.

## Footnotes

1
https://github.com/Roosje95/HEAT_gait-event-detection
