# Supplementary material for: Impact of the Marker Set Configuration on the Accuracy of Gait Event Detection in Healthy and Pathological Subjects
Source: Front Hum Neurosci. 2021 Sep 13;15:720699. doi: 10.3389/fnhum.2021.720699 (PMC8475178; doi:10.3389/fnhum.2021.720699)
Supplement: Supplementary file 1 [file Table_1.docx]

S1 - Participant groups based on foot orientation at mid-stance. Criteria were set based on the mean and SD of the sole angles in sagittal (IC) or frontal (TO) plane during midstance in the TD group, thresholds were chosen to allow for homogeneous distribution of participants between groups. Further information on grouping and calculation of the sole angles can be found on our GitLab page [https://github.com/Roosje95/HEAT_gait-event-detection]. TD: typical development, CP: cerebral palsy, SD: standard deviation, CI: confidential interval, NA: not applicable.

|  | **IC1** | **IC2** | **IC3** | **IC4** |
| --- | --- | --- | --- | --- |
| *Initial contact* | | | | |
| Criteria | > mean+2SD | mean-2SD ≤ mean+2SD | mean-10SD  ≤ mean-2SD | < mean-10SD |
| TD [N] | 62 | 0 | 0 | 0 |
| TD sole angle sagittal plane (◦) [median (CI95%)] | 17.8 (12.8:23.2) | NA | NA | NA |
| CP [N] | 51 | 25 | 42 | 38 |
| CP sole angle sagittal plane (◦) [median (CI95%)] | 9.8 (1.7:20.4) | -0.2 (-2.0:1.3) | -5.6 (-9.5:-2.4) | -15.5 (-34.4:-9.9) |

|  | **TO1** | **TO2** | **TO3** | **TO4** |
| --- | --- | --- | --- | --- |

| *Toe-off* | | | | |
| --- | --- | --- | --- | --- |
| Criteria | > mean+2SD | mean-2SD  ≤ mean+2SD | mean-4SD  ≤ mean-2SD | < mean-4SD |
| TD [N] | 0 | 11 | 30 | 21 |
| TD sole angle frontal plane (◦) [median (CI95%)] | NA | 4.3 (1.1:8.5) | -2.4 (-5.2:0.3) | -9.7 (-18.7:-5.4) |
| CP [N] | 15 | 55 | 36 | 50 |
| CP sole angle frontal plane (◦) [median (CI95%)] | 17.0 (13.3:38.7) | 5.6 (0.7:11.2) | -1.8 (-5.1:0.5) | -12.5 (-46.2:-6.6) |
