# Supplementary material for: Impact of the Marker Set Configuration on the Accuracy of Gait Event Detection in Healthy and Pathological Subjects
Source: Front Hum Neurosci. 2021 Sep 13;15:720699. doi: 10.3389/fnhum.2021.720699 (PMC8475178; doi:10.3389/fnhum.2021.720699)
Supplement: Supplementary file 2 [file Presentation_1.pptx]

## Slide 1
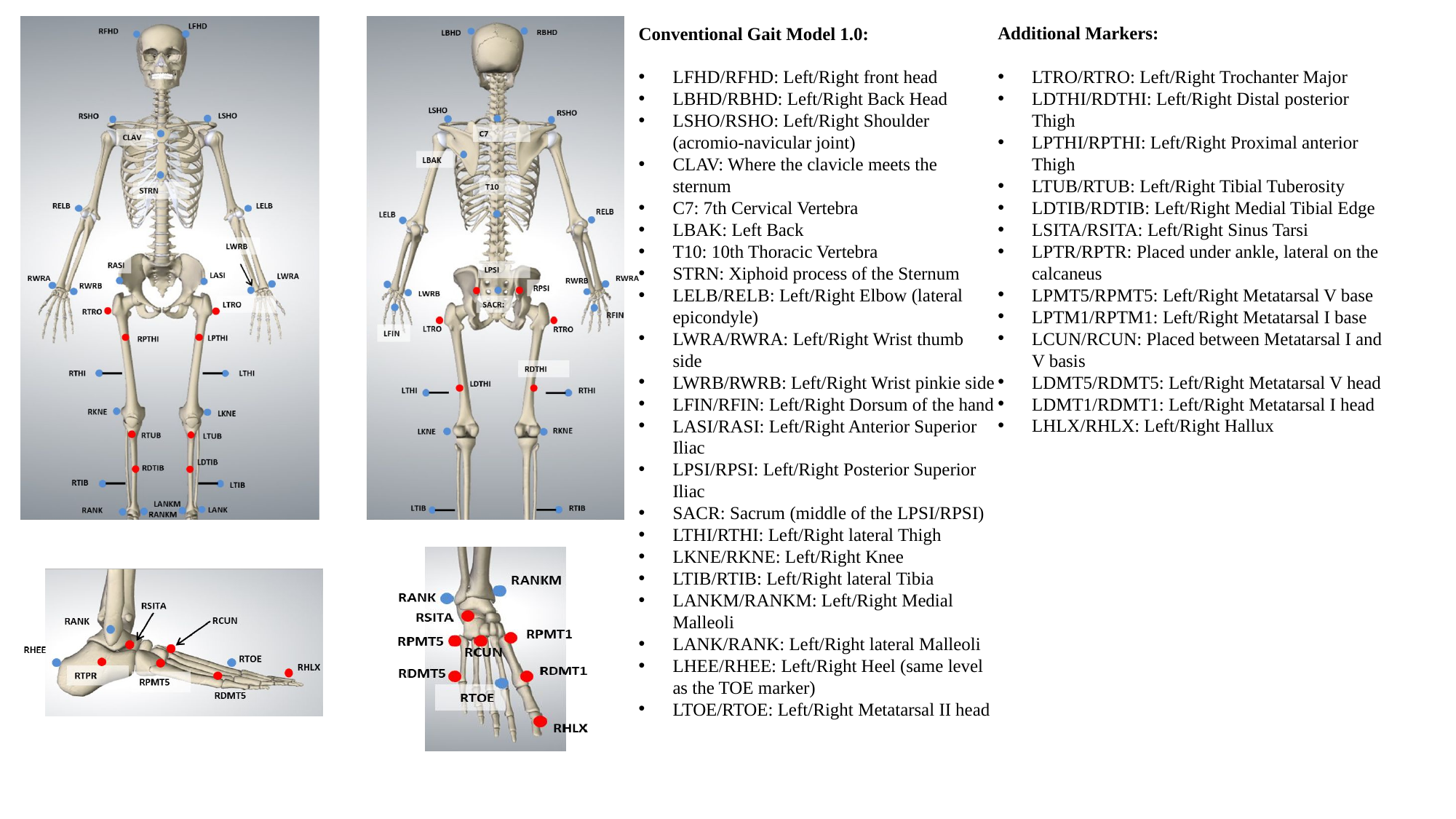

Conventional Gait Model 1.0:
LFHD/RFHD: Left/Right front head
LBHD/RBHD: Left/Right Back Head
LSHO/RSHO: Left/Right Shoulder (acromio-navicular joint)
CLAV: Where the clavicle meets the sternum
C7: 7th Cervical Vertebra
LBAK: Left Back
T10: 10th Thoracic Vertebra
STRN: Xiphoid process of the Sternum
LELB/RELB: Left/Right Elbow (lateral epicondyle)
LWRA/RWRA: Left/Right Wrist thumb side
LWRB/RWRB: Left/Right Wrist pinkie side
LFIN/RFIN: Left/Right Dorsum of the hand
LASI/RASI: Left/Right Anterior Superior Iliac
LPSI/RPSI: Left/Right Posterior Superior Iliac
SACR: Sacrum (middle of the LPSI/RPSI)
LTHI/RTHI: Left/Right lateral Thigh
LKNE/RKNE: Left/Right Knee
LTIB/RTIB: Left/Right lateral Tibia
LANKM/RANKM: Left/Right Medial Malleoli
LANK/RANK: Left/Right lateral Malleoli
LHEE/RHEE: Left/Right Heel (same level as the TOE marker)
LTOE/RTOE: Left/Right Metatarsal II head
Additional Markers:
LTRO/RTRO: Left/Right Trochanter Major
LDTHI/RDTHI: Left/Right Distal posterior Thigh
LPTHI/RPTHI: Left/Right Proximal anterior Thigh
LTUB/RTUB: Left/Right Tibial Tuberosity
LDTIB/RDTIB: Left/Right Medial Tibial Edge
LSITA/RSITA: Left/Right Sinus Tarsi
LPTR/RPTR: Placed under ankle, lateral on the calcaneus
LPMT5/RPMT5: Left/Right Metatarsal V base
LPTM1/RPTM1: Left/Right Metatarsal I base
LCUN/RCUN: Placed between Metatarsal I and V basis
LDMT5/RDMT5: Left/Right Metatarsal V head
LDMT1/RDMT1: Left/Right Metatarsal I head
LHLX/RHLX: Left/Right Hallux
